# Opioid Prescribing Patterns After Colorectal Resections in the United States of America

**DOI:** 10.7759/cureus.48890

**Published:** 2023-11-16

**Authors:** Uma R Phatak, Mukaila Raji, Lu Chen, Jacques G Baillargeon, Kuo Yong-Fang

**Affiliations:** 1 Department of Surgery, University of Texas Medical Branch, Galveston, USA; 2 Department of Internal Medicine, Division of Geriatrics and Palliative Medicine, University of Texas Medical Branch, Galveston, USA; 3 Department of Preventive Medicine and Population Health, University of Texas Medical Branch, Galveston, USA

**Keywords:** overprescribing, epidemic, pathway, gabapentinoid, opioid, colorectal

## Abstract

Background

The opioid epidemic is a significant source of morbidity and mortality in the United States of America. Minimizing opioid prescribing after operations has become an important component of post-operative care pathways. We hypothesized that opioid prescribing has decreased over time after colorectal resections.

Methods

This is a retrospective study from 2012 to 2019 using the Optum Clinformatics database (Eden Prairie, MN). We included patients aged 18 years or older who had an elective colorectal resection. Our primary outcome was the rate of opioid prescription at post-operative discharge. Secondary outcomes included the rates of gabapentinoid (GABA) prescribing post-operatively.

Results

Of 17,900 patients, the most common procedure was sigmoid colectomy (35%). Most procedures were open (N=10,626, 59.4%). The most common indication was benign disease (N=12,439, 69.5%). Post-operative opioid prescribing decreased from 64.4% in 2012 to 46.7% in 2019. In the adjusted model, the odds of post-operative opioid prescription were 37% lower in 2019 than in 2012 (OR, 0.63; 95% CI, 0.56-0.72; p<0.0001). At 60 days and one year post surgery, opioid prescribing decreased from 11.6% and 5.9% in 2012 to 7.2% and 5.2% in 2019 (p<0.0001). At 60 days, gabapentinoid prescribing increased from 2.3% in 2012 to 4.0% in 2019 (p=0.0016).

Conclusions

Our data show that opioid prescribing is common after colorectal surgery with an overall post-operative prescription rate of 55.8%. The modification of post-operative pathways to include guidance on opioid prescribing and non-opioid alternatives may curb opioid prescribing, decrease the number of new persistent opioid users, and decrease the number of opioids available for diversion.

## Introduction

In 2000, the Joint Commission on Accreditation of Healthcare Organizations (JCAHO) with support from the American Pain Society advocated for focusing efforts on better pain management, leading to the promotion of pain as the “fifth vital sign” [1]. JCAHO’s support of this position along with decreased regulation surrounding opioid prescription is thought to have fostered conditions for the opioid epidemic in the United States of America [2]. Although the United States of America represents about 5% of the world’s population, it accounts for about 70% of global opioid consumption [3]. Opioid overdose is the second leading cause of accidental death in the United States of America [4].

Around the same time that pain was dubbed the “fifth vital sign,” surgeons began publishing the results of enhanced recovery after surgery (ERAS) protocols, which include interventions that target patient physiology to improve outcomes and reduce the length of hospital stay [5-7]. Traditionally, elective colorectal surgery was associated with a hospital stay of at least eight days with a high complication rate (~30%) [8,9]. One of the reasons that patients remained in the hospital was post-operative ileus or delayed return of bowel function. An important component of these ERAS protocols is opioid minimization. Decreasing or avoiding opioids is associated with a decreased risk of ileus and a faster recovery of bowel function, ultimately resulting in a decreased length of hospital stay [2,10].

The purpose of this study was to assess the opioid prescribing practices in the United States of America after colorectal resection. In addition, we examined the incidence of new persistent opioid users after colorectal resection. We were particularly interested in whether minimally invasive surgery would be associated with a lower need for post-operative opioid prescriptions compared to open surgery. In the setting of a national opioid epidemic, these data will help to inform the creation or modification of discharge protocols for colorectal surgery.

## Materials and methods

Study design

We performed a retrospective cohort study using administrative health data from Optum’s deidentified Clinformatics Data Mart (Eden Prairie, MN) for 2012-2019. The Clinformatics Data Mart is a national, commercial, and Medicare Advantage health insurance claim database that covers at least 15 million patients per year across all age groups. We used enrollment, inpatient, outpatient, and pharmacy claim files. The University of Texas Institutional Review Board approved this study.

Study cohort

All patients who underwent a colorectal resection from 2012 to 2019 and were 18 years or older at the time of surgery were included. Colorectal resections were defined using the International Classification of Diseases, Ninth Revision, Clinical Modification (ICD-9-CM) and International Classification of Diseases, Tenth Revision, Clinical Modification (ICD-10-CM) codes based on the clinical classification software codes [11] for colorectal resection and the local excision of large intestine lesions. Patients were excluded if they were younger than 18 years, had other operations in the 12 months preceding their colorectal resection, or did not have continuous enrollment 12 months before the colorectal resection to seven days after hospital discharge.

Study outcomes

The primary outcome was opioid prescription after colorectal resection defined as at least one opioid pharmacy claim within three days before to seven days after hospital discharge. We repeated the analyses with the definition of post-operative opioid prescription as within 14 days before to 14 days after hospital discharge. Secondary outcomes included the rate of continuing opioid prescription after surgery among those receiving post-operative opioids. Continuous opioid use was defined as a prescription filled within 30 days of the end of the previous prescription’s supply duration. In addition, the initiation of gabapentinoid (GABA) prescription was examined among patients who did not have GABA prescriptions within three days before to seven days after hospital discharge.

Study covariates

Our primary independent variables of interest were the year of surgery and the type of surgery including approach (minimally invasive versus open). Other covariates included age, sex, the region of residence (the Northeast, South, Midwest, or West), selected comorbidities (anxiety, depression, drug use disorder, and alcohol use disorder), indication for surgery (benign versus malignant), and previous opioid and GABA prescription from four days before to 365 days before discharge. All comorbidities were identified in the 12 months before surgery by using the Elixhauser Comorbidity Index [12].

Statistical analyses

Demographic and clinical characteristics were summarized by post-operative opioid prescription. Bivariate and multivariable logistic regression models were used to assess factors associated with post-operative opioid prescription. The Kaplan-Meier method was used to estimate the rate of continuing opioid prescription and the rate of initiation of GABA prescription. Patients were censored at disenrollment, additional surgery, or one year after surgery, whichever occurred first. Bivariate and multivariable Cox proportional hazard models were used to estimate the hazard ratios (HR) of the effect on the year of surgery, the type of surgery, and other covariates on the continuation of opioid prescription and the initiation of GABA prescription. Proportional hazard assumptions were assessed by adding the interaction between the log of follow-up time and the covariate in the model. No violations of proportionality assumption were found [13]. All analyses were conducted using Statistical Analysis System (SAS) 9.4 (SAS Institute Inc., Cary, NC).

## Results

We identified 17,900 patients who met our criteria, of which 43.9% were male and the median age was 67 years (interquartile range {IQR}: 55-75) (Table 1). The most common indications for surgery were benign (69.5%), and most resections (59.4%) were open (Table 2).

**Table 1:** Patient characteristics ^1^Measured from day 4 prior to discharge to day 365 prior to surgery ^2^Measured in the 12 months prior to surgery GABA: gabapentin

	All (N=17,900)	Had opioid prescription at discharge (N=9,981)	No opioid prescription at discharge (N=7,919)
Age at surgery (years), median (Q1, Q3)	67 (55, 75)	60 (51, 70)	72 (64, 80)
Gender			
Female, N (%)	10,041 (56.1)	5,395 (54.1)	4,646 (58.7)
Male, N (%)	7,859 (43.9)	4,586 (45.9)	3,273 (41.3)
Region			
Northeast, N (%)	2,049 (11.4)	987 (9.9)	1,062 (13.4)
Midwest, N (%)	6,034 (33.7)	3,345 (33.5)	2,689 (34.0)
South, N (%)	5,622 (31.4)	3,344 (33.5)	2,278 (28.8)
West, N (%)	4,195 (23.4)	2,305 (23.1)	1,890 (23.9)
Prior opioid use^1^, N (%)	6,057 (33.8)	4,006 (40.1)	2,051 (25.9)
Prior GABA use^1^, N (%)	1,343 (7.5)	737 (7.4)	606 (7.7)
Surgery type			
Noncancer-related, N (%)	12,439 (69.5)	7,117 (71.3)	5,322 (67.2)
Cancer-related, N (%)	5,461 (30.5)	2,864 (28.7)	2,597 (32.8)
Surgery type			
Minimally invasive approach, N (%)	6,141 (34.3)	3,921 (39.3)	2,220 (28.0)
Open approach, N (%)	10,626 (59.4)	5,357 (53.7)	5,269 (66.5)
Undetermined approach, N (%)	1,133 (6.3)	703 (7.0)	430 (5.4)
Prior anxiety^2^, N (%)	2,499 (14.0)	1,489 (14.9)	1,010 (12.8)
Prior depression^2^, N (%)	2,749 (15.4)	1,566 (15.7)	1,183 (14.9)
Prior drug abuse^2^, N (%)	306 (1.7)	167 (1.7)	139 (1.8)
Prior alcohol abuse^2^, N (%)	361 (2.0)	220 (2.2)	141 (1.8)
Surgery year			
2012, N (%)	2,569 (14.4)	1,654 (16.6)	915 (11.6)
2013, N (%)	2,485 (13.9)	1,550 (15.5)	935 (11.8)
2014, N (%)	2,252 (12.6)	1,342 (13.4)	910 (11.5)
2015, N (%)	2,184 (12.2)	1,136 (11.4)	1,048 (13.2)
2016, N (%)	1,829 (10.2)	1,004 (10.1)	825 (10.4)
2017, N (%)	2,130 (11.9)	1,127 (11.3)	1,003 (12.7)
2018, N (%)	2,231 (12.5)	1,131 (11.3)	1,100 (13.9)
2019, N (%)	2,220 (12.4)	1,037 (10.4)	1,183 (14.9)

**Table 2:** Operation types and approaches

Operation	Minimally invasive approach, N (%)	Open approach, N (%)	Undetermined approach, N (%)	Total, N (%)
Left hemicolectomy	355 (1.98)	710 (3.97)	6 (0.03)	1,071 (5.98)
Right hemicolectomy	2,027 (11.32)	2,868 (16.02)	5 (0.03)	4,900 (27.37)
Sigmoidectomy	2,849 (15.92)	3,385 (18.91)	38 (0.21)	6,272 (35.04)
Resection of the rectum	189 (1.06)	300 (1.68)	981 (5.48)	1,470 (8.21)
Other colorectal resections	721 (4.03)	1,253 (7.00)	1 (0.01)	1,975 (11.03)
Local excision of large intestine lesion	0	2,110 (11.79)	102 (0.57)	2,212 (12.36)
	6,141 (34.31)	10,626 (59.36)	1,133 (6.33)	17,900

**Figure 1:**
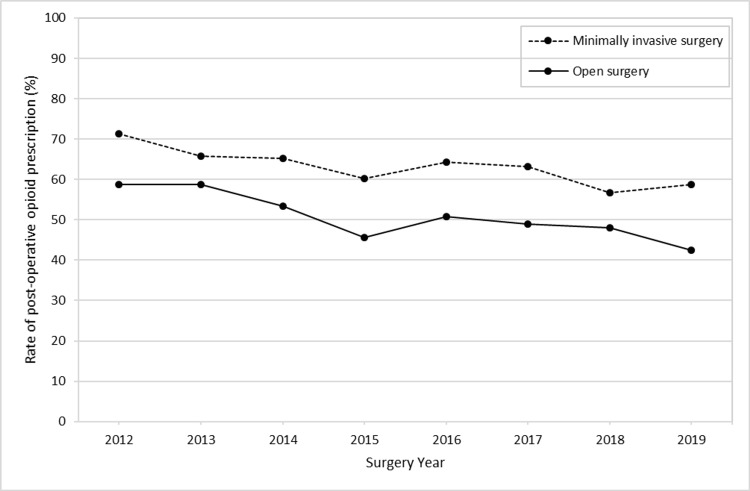
Proportion of patients with post-operative opioid prescription, stratified by surgery type

Of the total, 55.8% filled a prescription for opioids. These rates decreased from 64.4% in 2012 to 46.7% in 2019. Patients with minimally invasive surgery had a higher rate of post-operative opioid prescription compared to those with open surgery (63.9% versus 50.4%, p<0.0001). These rates decreased in both groups similarly (Figure 1). After adjusting for covariates, the odds of post-operative opioid prescription were 37% lower in 2019 compared to 2012 (OR, 0.63; 95% CI, 0.56-0.72; p<0.0001) and were 33% lower for those with open surgery compared to those with minimally invasive surgery (OR, 0.67; 95% CI, 0.63-0.72; p<0.0001). Other factors associated with post-operative opioid prescriptions were age at surgery, having surgery in the South or West regions, cancer as an indication for surgery, history of anxiety, and previous use of opioid (Table 2).

**Figure 2:**
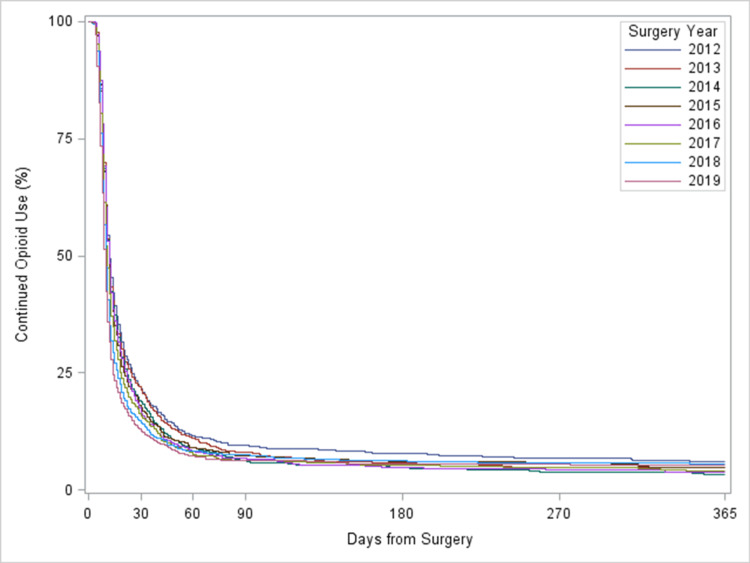
Proportion of patients with continued opioid utilization following colorectal surgery, stratified by surgery year

Of the 9,981 patients who received post-operative opioids, at 60 days and one year post surgery, the rate of new persistent opioid use decreased from 11.6% and 5.9% in 2012 to 7.2% and 5.2% in 2019 (p<0.0001) (Figure 2). The adjusted hazard of ceasing opioid use was 58% higher (HR, 1.58; 95% CI, 1.46-1.72; p<0.0001) in 2019 comparing to 2012. Therefore, over time, the rate of new persistent opioid decreased. Patients with open surgery were less likely to cease opioid use compared to those with minimally invasive surgery (HR, 0.64; 95% CI, 0.61-0.67; p<0.0001). Other factors associated with lower likelihood of ceasing opioids were residing in the South region, indication of cancer, comorbidity of anxiety, depression, drug use disorder or alcohol use disorder, and previous opioid and GABA use (Table 3).

**Table 3:** Adjusted and unadjusted relative odds of receiving an opioid prescription at discharge ^1^Measured from day 4 prior to discharge to day 365 prior to surgery ^2^Measured in the 12 months prior to surgery ^3^Undetermined approaches are included, but results are not reported GABA: gabapentin

Variables	Unadjusted			Adjusted		
	P-value	OR	95% CI	P-value	OR	95% CI
Age at surgery	<0.0001	0.54	0.53-0.56	<0.0001	0.56	0.54-0.57
Gender						
Female	Reference			Reference		
Male	<0.0001	1.21	1.14-1.28	0.4668	0.98	0.91-1.04
Region						
Northeast	Reference			Reference		
Midwest	<0.0001	1.34	1.21-1.48	0.0501	1.12	1.00-1.25
South	<0.0001	1.58	1.43-1.75	<0.0001	1.3	1.16-1.45
West	<0.0001	1.31	1.18-1.46	0.0011	1.21	1.08-1.36
Prior opioid use^1^	<0.0001	1.92	1.80-2.05	<0.0001	1.67	1.56-1.80
Prior GABA use^1^	0.4976	0.96	0.86-1.08	0.7362	1.02	0.90-1.16
Surgery type						
Noncancer-related	Reference			Reference		
Cancer-related	<0.0001	0.82	0.77-0.88	<0.0001	1.16	1.08-1.25
Surgery type^3^						
Minimally invasive approach	Reference			Reference		
Open approach	<0.0001	0.58	0.54-0.61	<0.0001	0.67	0.63-0.72
Prior anxiety^2^	<0.0001	1.2	1.10-1.31	0.0232	1.12	1.02-1.25
Prior depression^2^	0.1674	1.06	0.98-1.15	0.5431	0.97	0.88-1.07
Prior drug abuse^2^	0.6731	0.95	0.76-1.19	0.0009	0.65	0.50-0.84
Prior alcohol abuse^2^	0.0456	1.24	1.00-1.54	0.3869	1.11	0.88-1.40
Surgery year						
2012	Reference			Reference		
2013	0.1384	0.92	0.82-1.03	0.2451	0.93	0.82-1.05
2014	0.0006	0.82	0.73-0.92	0.0139	0.85	0.75-0.97
2015	<0.0001	0.6	0.53-0.67	<0.0001	0.67	0.59-0.76
2016	<0.0001	0.67	0.60-0.76	0.0006	0.79	0.69-0.90
2017	<0.0001	0.62	0.55-0.70	0.0002	0.78	0.69-0.89
2018	<0.0001	0.57	0.51-0.64	<0.0001	0.72	0.64-0.82
2019	<0.0001	0.48	0.43-0.54	<0.0001	0.63	0.56-0.72

**Figure 3:**
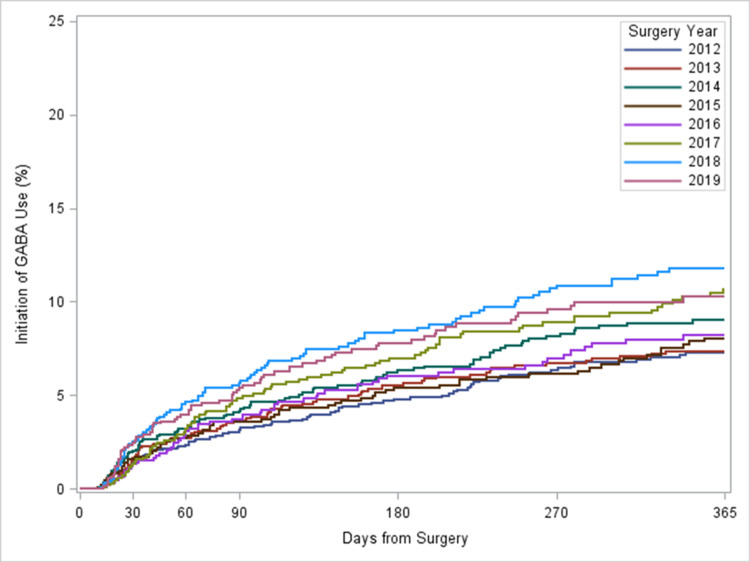
Proportion of patients with the initiation of GABA use following colorectal surgery, stratified by surgery year GABA: gabapentin

Of the 9,645 patients who had post-operative opioid prescription but did not have post-operative GABA prescriptions, at 60 days post surgery, the rate of the initiation of GABA increased from 2.3% in 2012 to 4.0% in 2019 (p=0.0016) (Figure 3). The unadjusted hazard of initiating GABA use was 52% higher (HR, 1.52; 95% CI, 1.12-2.07; p=0.0074) in 2019 compared to 2012. However, after adjusting for other covariates, there was no significant increase found in the hazard of initiating GABA use over the years. Patients with open surgery were more likely to initiate GABA use compared to those with minimally invasive surgery (HR, 1.24; 95% CI, 1.05-1.48; p=0.0138). Other factors associated with a higher likelihood of initiating GABA were older age, residing in the Midwest region, indication of cancer, comorbidity of alcohol use disorder, and previous opioid and GABA use (Table 4).

**Table 4:** Adjusted and unadjusted relative hazard of ceasing opioid utilization ^1^Measured from day 4 prior to discharge to day 365 prior to surgery ^2^Measured in the 12 months prior to surgery ^3^Undetermined approaches are included, but results are not reported GABA, gabapentin; HR, hazard ratio

Variables	Unadjusted			Adjusted		
	P-value	HR	95% CI	P-value	HR	95% CI
Age at surgery	0.9476	1	0.99-1.02	0.9097	1	0.99-1.02
Gender						
Female	Reference			Reference		
Male	0.0004	1.08	1.03-1.12	0.8356	1	0.95-1.04
Region						
Northeast	Reference			Reference		
Midwest	0.5568	0.98	0.91-1.05	0.743	0.99	0.92-1.06
South	<0.0001	0.78	0.73-0.84	<0.0001	0.81	0.75-0.87
West	0.1706	0.95	0.88-1.02	0.3009	0.96	0.89-1.04
Prior opioid use^1^	<0.0001	0.52	0.50-0.54	<0.0001	0.54	0.52-0.57
Prior GABA use^1^	<0.0001	0.58	0.53-0.63	<0.0001	0.67	0.61-0.73
Surgery type						
Noncancer-related	Reference			Reference		
Cancer-related	0.0015	0.93	0.89-0.97	<0.0001	0.84	0.80-0.89
Surgery type^3^						
Minimally invasive approach	Reference			Reference		
Open approach	<0.0001	0.68	0.65-0.71	<0.0001	0.64	0.61-0.67
Prior anxiety^2^	<0.0001	0.83	0.79-0.89	0.0075	0.92	0.86-0.98
Prior depression^2^	<0.0001	0.81	0.76-0.85	0.004	0.91	0.86-0.97
Prior drug abuse^2^	<0.0001	0.52	0.44-0.63	<0.0001	0.68	0.56-0.81
Prior alcohol abuse^2^	0.0006	0.77	0.67-0.90	0.0012	0.78	0.67-0.91
Surgery year						
2012	Reference			Reference		
2013	0.3996	1.03	0.96-1.11	0.7322	1.01	0.94-1.09
2014	0.0322	1.09	1.01-1.17	0.2442	1.05	0.97-1.13
2015	0.0387	1.09	1.00-1.18	0.0194	1.1	1.02-1.19
2016	0.0294	1.1	1.01-1.19	0.0133	1.11	1.02-1.21
2017	<0.0001	1.2	1.11-1.31	<0.0001	1.27	1.17-1.38
2018	<0.0001	1.3	1.20-1.41	<0.0001	1.46	1.34-1.58
2019	<0.0001	1.43	1.32-1.56	<0.0001	1.58	1.46-1.72

Finally, to assess the robustness of our findings, we conducted sensitivity analyses, repeating our analyses with the definition of post-operative opioid prescription within 14 days before to 14 days after hospital discharge. These findings were similar to the original results (Tables 3-5).

**Table 5:** Adjusted and unadjusted relative hazard of initiating GABA use ^1^Measured from day 4 prior to discharge to day 365 prior to surgery ^2^Measured in the 12 months prior to surgery ^3^Undetermined approaches are included, but results are not reported HR, hazard ratio; GABA, gabapentin

Variables	Unadjusted			Adjusted		
	P-value	HR	95% CI	P-value	HR	95% CI
Age at surgery	<0.0001	1.25	1.18-1.33	<0.0001	1.16	1.09-1.24
Gender						
Female	Reference			Reference		
Male	<0.0001	0.66	0.57-0.78	0.3064	0.92	0.77-1.08
Region						
Northeast	Reference			Reference		
Midwest	0.1829	1.23	0.91-1.67	0.0335	1.4	1.03-1.90
South	0.0348	1.39	1.02-1.88	0.1672	1.24	0.91-1.69
West	0.0682	1.34	0.98-1.84	0.1219	1.29	0.93-1.77
Prior opioid use^1^	<0.0001	2.19	1.88-2.56	<0.0001	1.62	1.38-1.91
Prior GABA use^1^	<0.0001	25.08	21.47-29.29	<0.0001	21.62	18.35-25.48
Surgery type						
Noncancer-related	Reference			Reference		
Cancer-related	<0.0001	1.51	1.29-1.78	<0.0001	1.55	1.31-1.84
Surgery type^3^						
Minimally invasive approach	Reference			Reference		
Open approach	<0.0001	1.4	1.19-1.66	0.0138	1.24	1.05-1.48
Prior anxiety^2^	0.001	1.39	1.14-1.69	0.6871	1.05	0.84-1.30
Prior depression^2^	<0.0001	1.68	1.40-2.02	0.4239	1.09	0.89-1.33
Prior drug abuse^2^	<0.0001	3.15	2.16-4.61	0.0954	1.4	0.94-2.08
Prior alcohol abuse^2^	0.0043	1.83	1.21-2.78	0.0296	1.6	1.05-2.45
Surgery year						
2012	Reference			Reference		
2013	0.705	1.06	0.79-1.42	0.7309	1.05	0.78-1.42
2014	0.0953	1.28	0.96-1.71	0.1036	1.27	0.95-1.71
2015	0.5445	1.1	0.80-1.52	0.8909	0.98	0.71-1.35
2016	0.4107	1.15	0.83-1.60	0.9488	0.99	0.71-1.39
2017	0.0101	1.47	1.10-1.98	0.2158	1.21	0.89-1.64
2018	0.0002	1.72	1.29-2.29	0.089	1.3	0.96-1.75
2019	0.0074	1.52	1.12-2.07	0.9882	1	0.73-1.36

## Discussion

In this study of patients who underwent colorectal resections between 2012 and 2019, we found that overall, more than 50% of patients were given prescriptions for post-operative opioids. The number of minimally invasive procedures was lower than traditional open procedures. However, the patients who received minimally invasive surgery had higher odds of being prescribed opioids compared to patients who received an open operation. This result was unexpected. Over time though, the odds of receiving an opioid prescription decreased. We did find that patients who underwent minimally invasive surgery had a shorter duration of use of post-operative opioids and were less likely to initiate GABA analgesics.

It has been reported that up to 71% of post-operatively prescribed opioids go unused [14]. Some have sought to refine guidance on post-operative opioid prescribing to reduce the amount available for diversion in the community. A multi-institutional study on opioid prescribing after 21 different urologic procedures found that the implementation of a discharge prescribing guideline decreased opioid prescriptions after surgery by about 50% [15]. Similarly, a multispecialty intervention focused on patients and providers resulted in a significant increase in the number of patients discharged after surgery without an opioid prescription [16]. In addition, in this study, disparities in prescribing by sex or race disappeared post intervention. The results also showed that decreased prescribing did not lead to increased requests for refills.

Studies of enhanced recovery after surgery (ERAS) protocols have long touted the benefit of multimodal analgesia in decreasing the inpatient usage of opioids [2]. However, few ERAS studies have reported on opioid prescribing at discharge. A single-center before-and-after study of an ERAS for colorectal surgery found that 72% of patients who had minimal to no opioid requirements during their hospital stay still received opioids on discharge. They showed an increase in opioid-free analgesia during the inpatient stay after the ERAS intervention; however, this did not translate to decreased opioid prescriptions at discharge [17]. They concluded that more attention needed to be given to discharge prescribing practices. A systematic review of ERAS studies with opioid-free analgesia after “major surgery” found only eight randomized studies that targeted prescribing at hospital discharge [18]. Another interesting finding of this review is that only about 5% of the included studies were conducted in North America, a global hotspot in terms of the opioid epidemic.

Our results showed a decrease over time in new persistent opioid use after colorectal surgery. A retrospective study of patients in Canada who had curative intent surgery for cancer revealed an 8% rate of new persistent opioid use [19]. Among the new persistent users, there were higher rates of opioid overdose, increased healthcare utilization, and worse overall survival. A similar study of patients in the United States of America who had surgery for cancer revealed a new persistent opioid use rate of 10.4% [20]. In this study, receipt of adjuvant chemotherapy and radiation were associated with increased risks of new persistent opioid use. In a study of opioid-naïve patients undergoing gynecologic surgeries, perioperative opioid use was associated with an increased risk of becoming a new persistent opioid user [21]. These studies show that perioperative opioid use after any procedure can lead to new persistent use, and this can be associated with poor outcomes.

There are limitations to this study. First, because this investigation relied on a commercial health insurance claim database, these findings cannot be generalizable to uninsured or older Americans with Medicare fee-for-service coverage. Second, we were not able to identify who wrote the opioid prescription: attending, resident, or other mid-level provider. Therefore, confounding associated with differences in opioid prescribing by level of training cannot be ascertained. The amounts of opioids received as an inpatient were also not available. Therefore, we cannot draw conclusions on the post-operative prescribing of opioids based on inpatient usage. Prescription claim data also do not capture information on drugs obtained outside of the health plan. Further, our data do not include information on race/ethnicity or socioeconomic status. Lastly, the claim database does not contain information regarding the use of ERAS pathways at individual institutions. The results of this study cannot be used to determine the success or failure of ERAS pathways.

## Conclusions

Our results show that opioid prescribing after colorectal resections is prevalent across the United States of America. In addition, there was a higher rate of opioid prescribing after minimally invasive procedures compared to open procedures, which was surprising. These data show that more attention needs to be paid toward modifying discharge protocols to include guidance on post-operative opioid prescribing and the consideration of non-opioid alternatives. In addition, there needs to be a national focus on setting guidelines for post-operative opioid prescribing to decrease the number of opioids available for diversion and decrease the risk of creating new persistent users.
